# Safety and effectiveness of the Kaiser Permanente early-onset neonatal sepsis calculator in Qatar

**DOI:** 10.3389/fped.2025.1637914

**Published:** 2025-11-21

**Authors:** Anvar Paraparambil Vellamgot, Sajid Thyvilayil Salim, Khalil Salameh, Sudheer Babu Kurunthattilthazhe, Abdurahiman Elikkottil, Lina Habboub, Rajesh Pattuvalappil, Biny Elizabeth Joseph

**Affiliations:** 1 Department of Neonatology, Al Wakra Hospital, Hamad Medical Corporation (HMC), Al Wakrah, Qatar; 2 Department of Neonatology, Women’s Wellness and Research Centre, Hamad Medical Corporation, Doha, Qatar; 3 Department of Pediatrics and Neonatology, Al Khor Hospital, HMC, Al Khor, Qatar

**Keywords:** stewardship, newborn, sepsis, sepsis-calculator, safety

## Abstract

**Introduction:**

Early-onset neonatal sepsis is a significant cause of neonatal morbidity and mortality worldwide. Although sepsis rates are declining, neonatal antibiotic use remains high. The early-onset sepsis risk calculator, endorsed by the American Academy of Pediatrics, is one of three evidence-based methods for identifying at-risk babies. This study retrospectively compared the calculator to the existing categorical approach.

**Objectives:**

The primary objective was to compare the effectiveness of the calculator with the existing categorical approach in identifying sepsis cases within the first 12 h of life. Secondary aims included describing the calculator's recommendations and identifying predictors of missed cases.

**Methods:**

We retrospectively analyzed cases of early-onset sepsis in late-preterm and term infants born in Qatar between 2015 and 2022. We compared the calculator's predicted effectiveness to the current categorical approach.

**Results:**

Among 179,147 live births, 157 cases were identified (0.88 per 1,000). Of 105 cases (≥34 weeks), the calculator recommended antibiotics at birth in 37.1% (95% CI: 27.8%–46.4%) compared to 52% (42.9%–62.0%) by the categorical approach (*p* < 0.01), missing 16 cases. By 12 h, it identified 58.1% vs. 72.5% by the categorical approach (*p* < 0.01), missing 15 cases. Overall, the calculator missed six more cases than the categorical approach.

**Conclusions:**

The calculator identified fewer cases and delayed treatment in some neonates compared to current practice. It should be used cautiously, tailored to local risks and clinical context, with close postnatal monitoring. Additional local prospective studies are necessary to improve EOS management and reduce unnecessary antibiotic use.

## Introduction

Early-onset neonatal sepsis (EOS) is a bacterial infection of the bloodstream or cerebrospinal fluid that occurs within 72 h of birth, with a 3% mortality rate among late preterm and term neonates (1). Key risk factors for EOS include premature rupture of membranes (ROM), maternal fever, prematurity, prolonged labor, and colonization with Group B Streptococcus (GBS) or other pathogenic bacteria (2). GBS and E. coli are the leading organisms (2). The global incidence of EOS varies widely, ranging from 0.5–0.79 per 1,000 live births in high-income countries (3, 4) and 3–24 per 1,000 in low- and middle-income countries (5). Developed countries, such as the United States, have reported declining EOS rates, primarily due to the widespread use of maternal screening and intrapartum prophylaxis for GBS (1). The implementation of universal GBS screening and the Centers for Disease Control and Prevention (CDC) guidelines in the USA resulted in 14%–24% of newborns undergoing blood tests and 5%–12% receiving antibiotics, while the EOS incidence remained below 0.1% (6, 7).

Neonatal antibiotic use has been associated with a higher risk of asthma, allergy, and autoimmune diseases (8–10). Other potential drawbacks include longer hospital stays, separation from the mother, and increased costs. The American Academy of Pediatrics (AAP) recommends three evidence-based strategies for the secondary prevention of EOS (2). These are the multivariate risk prediction model, categorical risk assessment, and reserving antibiotics for infants showing clinical signs. The National Institute for Health and Care Excellence (NICE) UK suggests using a combination of clinical indicators and risk factors, recommending antibiotics if multiple criteria or red flag signs are present, while advocating for close monitoring in lower-risk cases (11). Other organizations, such as the Canadian Pediatric Society, employ similar risk- and sign-based algorithms, structured assessments, and ongoing clinical evaluations (12).

The Early-Onset Sepsis risk Calculator (EOSCAL), designed by the Kaiser Permanente (KP) group in the US, is a multivariate model for predicting the EOS risk in neonates (13). It is accessible online at https://neonatalsepsiscalculator.kaiserpermanente.org/InfectionProbabilityCalculator.aspx.

EOSCAL derives the Sepsis Risk Score (SRS) at birth by modifying the population risk with intrapartum risk factors specific to the mother-infant pair; it then incorporates the infant's clinical status during the first 12 h to compute the final SRS. Although the SRS computation algorithm is evidence-based, the clinical management recommendations (based on the SRS) are based on the consensus of the original researchers. The original model (2017 version) was based on data collected prior to the implementation of universal maternal GBS screening across KP hospitals. The authors of EOSCAL have recently updated the calculator (2024 version) based on more recent prospective data and recommend using the updated calculator in the context of universal GBS screening.

They recommended intravenous antibiotics for SRS ≥ 3, blood culture for SRS between 1 and <3, and routine care for SRS < 1. The implementation of EOSCAL in KP hospitals resulted in a 50% reduction in antibiotic use without increasing the incidence of missed EOS or readmissions (13). Multiple centers have consistently reported a 45%–50% reduction in antibiotic use following the implementation of the calculator (6, 14).

The AAP policy has endorsed EOSCAL for managing EOS in infants greater than 34 weeks' gestation (2). However, some reports have raised concerns about the risk of delayed and missed treatment for babies with EOS. Scott et al. (15) reported that the use of EOSCAL missed 35% of EOS cases, compared to 3% with the Australian guidelines. In a systematic review including 75 EOS cases identified by the NICE recommendation, Pettinger et al. (2019) (16) estimated that at least 14 (18%) cases would be missed if EOSCAL was used.

Since mid-2018, Qatar has employed the universal GBS screening strategy for pregnant mothers. The overall incidence of GBS EOS in Qatar is 0.58/1,000, and no significant trend has been observed since 2015 (17). To manage neonates at risk for EOS, the hospitals in the country currently follow the categorical approach proposed by the CDC (18), resulting in high antibiotic use. A recent quality improvement study from Qatar (19) observed that 11% of all liveborn babies received early antibiotics. The same study reported a 60% reduction in antibiotic use among term, well-appearing chorioamnionitis-exposed babies when EOSCAL was applied, without missing any actual EOS cases. Although EOSCAL use significantly impacts patient care, flow, and cost reduction, the possibility of missing and delaying treatment is a concern. Our study aimed to estimate the proportion of EOS cases that would have been correctly identified by retrospectively applying EOSCAL to a cohort of culture-proven EOS cases in Qatar.

Our study aimed to estimate the proportion of culture-proven EOS cases in Qatar that would have been correctly identified by retrospectively applying EOSCAL.

## Materials and methods

### Objectives

#### Primary objective

To estimate the proportion of EOS cases that would have been identified during the first 12 h of age by retrospectively applying the EOSCAL, compared to the proportion identified by the existing practice (CDC Approach).

#### Secondary objectives

1. To describe the projected EOSCAL recommendation upon initial assessment and over the first 12 h.
2. To describe Variables, if any, that would predict the cases missed by EOSCAL.

### Study design, setting, and population

This study was a retrospective chart review targeting neonates born at three main Hamad Medical Corporation (HMC) hospitals in Qatar: the Women's Wellness and Research Centre, Al Wakra Hospital, and Al Khor Hospital. The study included infants who developed EOS within the first 72 h of life, born between June 1, 2015, and December 31, 2022. Given the rarity of the condition and the potential for incomplete data, a seven-year study period was selected to ensure adequate case capture. HMC serves as the principal healthcare provider in Qatar, accounting for over 95% of all childbirths within the country. The hospitals under HMC employ Cerner Millennium as the electronic health record documentation system.

A data specialist in the medical records department reviewed records of all live-born infants during the study period. The specialist then identified neonates for whom blood or cerebrospinal fluid cultures were ordered within the first 72 h of life. Infants with positive culture results were selected for further analysis. For these cases, detailed maternal and neonatal data were extracted from the electronic medical record system.

### Inclusion and exclusion criteria

The study included late preterm (34–36 weeks) and term infants born at HMC hospitals between 2015 and 2022 who developed EOS within the first 72 h of life. The exclusion criteria included a positive blood culture after 72 h of age, growth of a non-pathogenic bacterium considered as contamination, babies born outside the three HMC hospitals, gestational age less than 34 weeks, and cases with incomplete data.

### Variables and outcomes

Maternal data related to infection risk factors, such as GBS status, intrapartum fever, chorioamnionitis, duration of ROM, timing, and type of intrapartum antibiotics received, were collected.

Neonatal data included the total number of live births and EOS during the period, baseline demographics, perinatal depression, onset and nature of presenting symptoms, if any, during the first 72 h, timing and type of antibiotics administered, type of organism, and respiratory support required. Details of clinical status during the first 72 h of life, with special emphasis on the first 12 h, were collected.

Babies were classified as well-appearing, equivocal, or clinically ill based on the following definitions (13).

**Clinical illness**: was defined as any one of the following: a persistent need for nasal continuous positive airway pressure, heated humidified high-flow nasal cannula, or mechanical ventilation outside of the delivery room; hemodynamic instability requiring vasoactive drugs; neonatal encephalopathy or perinatal depression, manifested by seizures or an Apgar score at 5 min less than 5; or the need for supplemental oxygen for more than 2 h to maintain oxygen saturations above 90% outside of the delivery room.

**Equivocal state**: was defined as a persistent physiologic abnormality lasting more than 4 h, which may include tachycardia with a heart rate over 160 beats per minute, tachypnea with a respiratory rate over 60 breaths per minute, temperature instability with a temperature greater than 100.4°F or less than 97.5°F, or respiratory distress—such as grunting, flaring, or retracting—that does not require supplemental oxygen. Alternatively, the presence of two or more physiologic abnormalities (as listed above) lasting for more than 2 h also met this definition.

**Well appearing** was defined as the absence of any persistent physiologic abnormalities. EOSCAL was applied at birth and at 12 h of age.

The calculator was accessed at: https://neonatalsepsiscalculator.kaiserpermanente.org/InfectionProbabilityCalculator.aspx.

Since universal GBS screening was not in use in Qatar before 2018, and our data collection period spanned from 2015–2022, we used the 2017 version of EOSCAL. The parameters entered into the calculator included the baseline population risk of EOS (equal to 1 in 1,000), gestational age, duration of rupture of membranes, peak intrapartum temperature, and GBS carrier status of the mother.

The primary outcome was the number of EOS cases identified by EOSCAL by 12 h of age, compared to the 2010 CDC guideline (18). Cases for whom antibiotics or blood culture were not recommended, even at the end of 12 h, were considered as missed cases. The secondary outcomes included the details of the projected EOSCAL recommendations and predictors for missing cases.

### Statistical analysis

Statistical analysis was performed using the statistical package SPSS 22.0 (SPSS Inc., Chicago, IL). The number of EOS cases per 1,000 live births was calculated.

The proportions of EOS cases identified using the projected EOSCAL and categorical approaches were estimated with 95% confidence intervals. Descriptive statistics were used to summarize all demographic data, risk factors, and outcome data. The results were reported as means and standard deviations (SD) or frequencies and percentages, depending on the type of data. The McNemar test was used to compare the proportion of EOS cases identified by EOSCAL and categorical approaches. A logistic regression was used to assess the predictors of cases missed by EOSCAL. All presented *P* values are two-tailed, and a *P* value less than 0.05 was considered statistically significant.

## Results

Table 1 shows the baseline data. Between July 2015 and December 2022, among 179,147 live births, there were 157 cases of early-onset sepsis (EOS), resulting in an incidence rate of 0.88 per 1,000 live births. The study included 105 EOS cases born at 34 weeks of gestation or later. Of these, 44.9% were male and 58.1% were female; 40% were Qatari and 60% non-Qatari. The mean birth weight was 3,203 grams (±460), and the mean gestational age was 38.4 weeks (±1.6). Most infants were between 37 and 40 weeks' gestation. Group B Streptococcus was the predominant organism (69.5%), followed by E. coli (7.6%). The mortality rate among the included cases was 7.6%.

**Table 1:** Baseline data.

Variable		Overall, from July 2015 to December 2022
Total live births		1,79,147
Total EOS		157
Incidence of EOS		0.88/1,000 live births
Included cases (34 weeks or more)		105
Male, No (%)		44 (44.9%)
female, No (%)		61 (58.1%)
Qatari, No (%)		42 (40%)
Non-Qatari, No (%)		63 (60%)
Birth weight in grams, mean (SD)		3,203.41 (± 460.27)
Gestational age in weeks, mean (SD)		38.4 (1.6)
Gestational age distribution, No (%)	34–36 weeks	14 (13.3%)
	37–41 weeks	91 (86.7%)
Cultured organism, No (%)	GBS	73 (69.5%)
	E Coli	8 (7.6%)
	Enterococci	5 (4.8%)
	Staphylococcus aureus	3 (2.9%)
	Haemophilus influenza	3 (2.9%)
	Others	13 (12.4%)
Mortality		8 (7.6%)

EOS, early onset sepsis; GBS, group B streptococcus.

**Figure 1:**
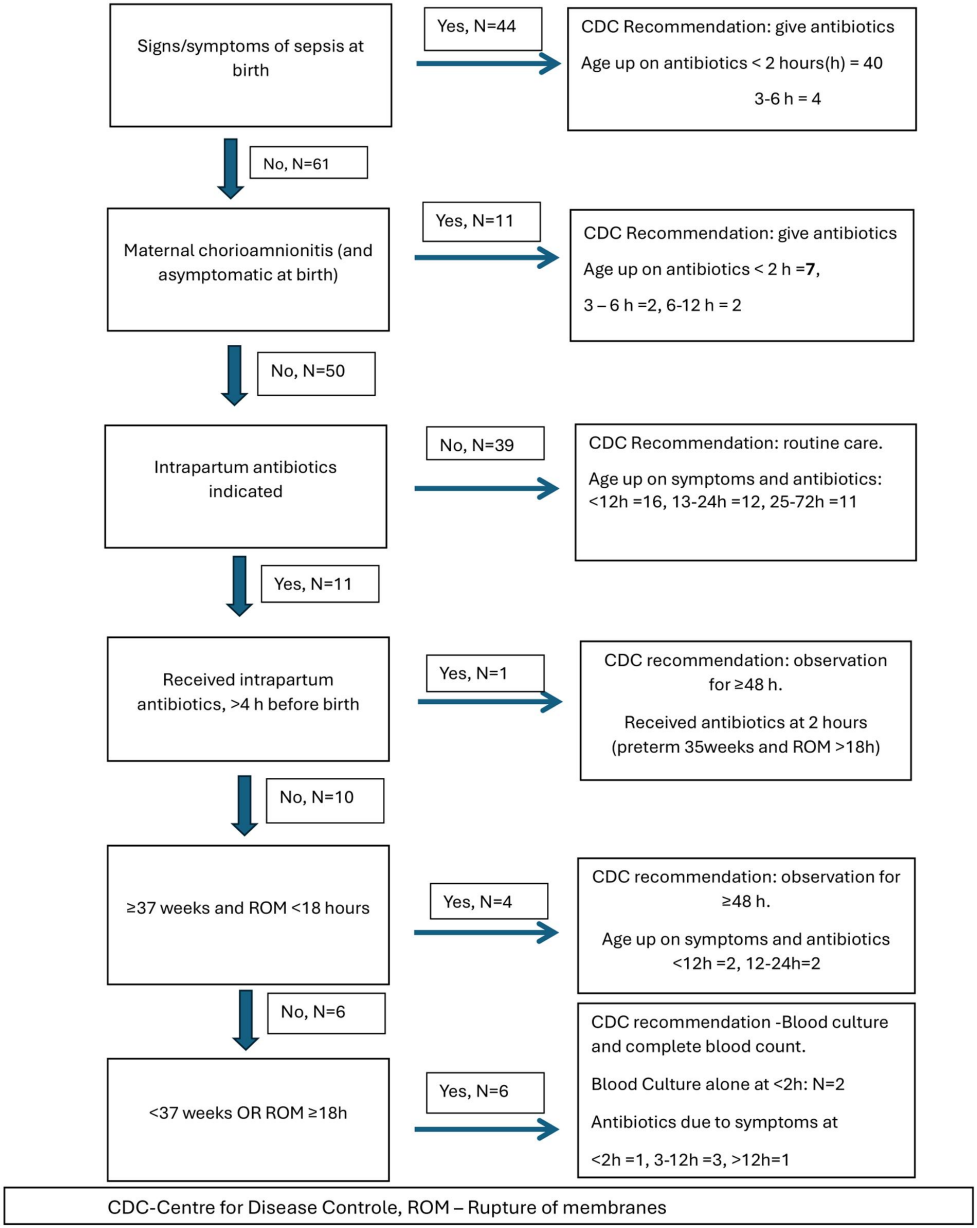
CDC algorithm for risk categorization and timing of management in affected neonates.

The categorization and timing of management, according to the CDC algorithm (18), are shown in Figure 1.

Peripartum risk factors and neonatal clinical status are detailed in Table 2. Premature rupture of membranes beyond 18 h before delivery occurred in 13.3% of cases. The GBS carrier status was unknown for 54.3% of mothers, and 12.4% were confirmed carriers. Approximately 22% of mothers had an intrapartum temperature ≥38°C, and 28.6% had chorioamnionitis. Although antibiotics were indicated for 41.9% of mothers, only 6.7% received adequate intrapartum antibiotics. Among 17 mothers who received intrapartum antibiotics more than 2 h before delivery, 15 received broad-spectrum antibiotics, which included a combination of ampicillin or ceftriaxone with gentamicin.

**Table 2:** Peripartum risk factors and neonatal clinical status.

Parameter		Number (%)
No identifiable prenatal factors for sepsis (except for unknown GBS status)		43 (40%)
Duration of rupture of membrane	0 h	16 (15.2%)
	1–18 h	75 (71.4%)
	>18 h	14 (13.3%)
GBS carrier status	Positive	13 (12.4%)
	Negative	35 (33.3%)
	Unknown	57 (54.3%)
Highest Intrapartum temperature of 38°C and more,		23 (21.9%)
Chorioamnionitis		30 (28.6%)
Intrapartum antibiotics indicated		44 (41.9%)
No antibiotics/any antibiotics < 2 h before delivery,		88 (83.8%)
GBS-specific antibiotics > 2 h before delivery		2 (1.9%)
Broad-spectrum antibiotics 2–3.9 h before delivery		5 (4.8%)
Broad-spectrum antibiotics >4 h or after delivery		10 (9.5%)
Clinical status during the first hour	Asymptomatic and well-appearing	59 (56.2%)
	Equivocal	2 (1.9%)
	Clinical illness	44 (41.9%)
Number of initially asymptomatic babies who became symptomatic within 72 h		50/59 (84.8%)
Age of onset of symptoms- Cumulative No (%)	First hour of life	44 (41.9%)
	Within 6 h	63 (60%)
	Within 12 h	71 (67.6%)
	Within 24 h	85 (80.9%)
	Within 72 h	96 (91.5%)
	Never symptomatic	9 (8.5%)
No. of symptomatic babies with symptoms not defined as clinical illness or equivocal illness		12 (11.4%)
Clinical status at 12 h of age	Well-appearing	34 (32.3%)
	Equivocal	2 (1.9%)
	Clinical illness	69 (65.7%)

GBS, group B streptococcus.

During the first hour of life, 56.2% of infants were well-appearing, 1.9% had equivocal status, and 41.9% showed clinical illness. Symptom onset was gradual, occurring in 41.9% within the first hour, rising to 67.6% by 12 h, and 91.5% by 72 h. About 11.4% exhibited symptoms such as hypoactivity or poor sucking and were not classified as clinical or equivocal illness. Notably, 8.5% of babies remained asymptomatic throughout.

By 12 h of age, 58.1% were categorized as clinically ill, 40% as well-appearing, and 1.9% as equivocal.

At birth, 54.3% of neonates had an SRS below 1, indicating routine care, while 37.1% had an SRS of 3 or higher, warranting blood cultures and antibiotics (Table 3). By 12 h, the proportion with SRS ≥ 3 increased to 58%. Correspondingly, those with SRS < 1 decreased to 33%.

**Table 3:** Distribution of SRS at birth and 12 h of age.

SRS (per 1,000 LB)	At Birth	By 12 h	SRS recommendation
<1	57 (54.3%)	42 (40%)	Routine care
1 to <3	9 (8.6%)	9 (8.6%)	Blood Culture and observation
3 and more	39 (37.1%)	61 (58%)	Blood Culture and Antibiotics

SRS, sepsis risk score.

Table 4 shows the comparative efficacy of EOSCAL and CDC recommendations in identifying EOS cases at birth and at 12 h of age. At birth, the EOSCAL recommended antibiotics in 37.1% (95% CI, 27.8%–46.4%) of cases, significantly fewer than the CDC guidelines, which recommended antibiotics in 52% (95% CI, 42.9%–62.0%) (*p* < 0.01). This resulted in the delayed identification of 16 additional cases by EOSCAL, compared to the CDC recommendation. By 12 h, EOSCAL's detection rate improved to 58.1% (95% CI 48.6%–67.5%) but remained significantly lower than the CDC recommendation of 72.5% (95% CI 63.9%–80.9%) (*p* < 0.01), resulting in missed or delayed antibiotic treatment in 15 cases. However, considering 9 cases for whom SRS recommended blood culture, the overall number of missed cases would have been 6.

**Table 4:** Cases identified and missed/delayed by EOSCAL vs. CDC recommendations.

Age	Comparison of cases identified by SRS Vs. CDC recommendations			Cases who received antibiotics, No (%)	Additional number of cases missed/delayed by SRS, when compared to CDC recommendation
	Cases for whom SRS recommended antibiotics, No (%) (95% CI)	Cases for whom CDC recommended antibiotics, No (%) (95% CI)	*P*-Value (McNemar test)		
At birth	39 (37.1%)	55 (52%)	*P* < 0.01	48 (45%) (within first 2 h of life)	16
	(27.8%–46.4%)	(42.9%–62.0%)			
By 12 h	61 (58.1%)	76 (72.5%)	*P* < 0.01	75 (71.5%)	15
	(48.6%–67.5%)	(63.9%–80.9%)			

EOSCAL, early onset sepsis risk calculator; CDC, centre for disease control.

There were eight deaths (7.6%), and all deaths occurred after 12 h of age. Among the eight babies who expired, EOSCAL recommended antibiotics for two and blood culture for another two at birth. CDC recommended antibiotics for all four of them at birth. By 12 h, neither EOSCAL nor CDC had recommended antibiotics for three infants, who subsequently died between 18 and 32 h of age.

Logistic regression was used to identify predictors for the missed cases by EOSCAL but not by the categorical approach of CDC (Table 5). All the evaluated variables—including gender, gestational age, birth weight, GBS EOS, chorioamnionitis, mode of delivery, ROM > 18 h, being symptomatic by 1 h, or being asymptomatic during the first 12 h—were not significant.

**Table 5:** Characteristics of missed EOS cases (*N* = 15)—logistic regression.

Variable	*p*-value	Adjusted OR	95% CI
(Categorical variable coding –			
1 = present,			
0 = absent (ref)			
female gender	0.85	1.13	(0.32, 3.98)
Prematurity	0.99		
Birth weight	0.38	0.99	0.98 -1.01
GBS EOS	0.17	2.7	(0.66, 10.97)
Chorioamnionitis	0.66	0.72	(0.17, 3.10)
Normal vaginal delivery	0.18	2.99	(0.60, 14.90)
ROM > 18 h	0.14	5.94	(0.56, 63.28)
Symptomatic by 1 h	0.66	1.44	(0.28, 7.28)
Asymptomatic before 12 h of age	0.99	0.98	(0.18, 5.25)

EOS, early onset sepsis; GBS, group B streptococcus; ROM, rupture of membranes.

## Discussion

This study observed an EOS incidence of 0.88/1,000 live births, with a predominance of GBS, aligning with reports from high-income countries (3, 4). The observed mortality of 7.6% among late preterm and term babies with EOS exceeded the reported rate of 3% in developed countries (20).

Notably, 40% of babies had no identifiable prenatal risk factors other than unknown GBS status (term gestation, no indication for intrapartum antibiotics, no chorioamnionitis, no maternal fever or rupture of membranes beyond 18 h). These babies were identified based on clinical signs or symptoms at various time points, from birth to 72 h. If the CDC recommendation (18) was applied, 42% of mothers would require intrapartum antibiotics. However, more than 75% of them did not receive adequate intrapartum antibiotics, mostly due to the late appearance of risk factors, such as fever.

More than 50% of the babies were asymptomatic during the first hour. Even at 12 h of age, 40% of the babies continued to appear well. Moreover, some babies with symptoms like poor sucking or hypoactivity would have been misclassified as well-appearing. Consequently, EOSCAL would have recommended antibiotics to only 37% (95% CI, 27.8%–46.4%) during the first hour and to 58% (95% CI, 48.6%–67.5%) by 12 h. This would be significantly lower (*p* < 0.01) than the cases identified by the CDC's recommendation of 52% (95% CI 42.9%–62.0%) during the first hour and 72.5% (95% CI 63.9%–80.9%) by 12 h of age. Compared to the CDC recommendation, EOSCAL would have delayed antibiotics in 16 additional cases during the first hour and 15 cases by 12 h of age. Excluding 9 babies for whom the calculator recommended blood culture without antibiotics, six additional cases would have been missed by EOSCAL at 12 h.

Following the publication of the original recommendation (13), several studies have confirmed the efficacy of EOSCAL in reducing the blood culture rate, antibiotic use, and hospital stay (6, 14). The AAP has also recommended it as an evidence-based tool for risk stratification and management of EOS. The recently updated EOSCAL model (21) has a sensitivity of 0.80 (95% CI 0.68–0.89). They also reported that the use of EOSCAL did not increase the incidence or mortality of EOS. Yoshida et al. (2022) (22) conducted a prospective observational study in 26 NHS neonatal units over a 12-month period, comparing EOSCAL with NICE guidelines. There was no statistically significant difference in the number of culture-proven EOS cases missed by EOSCAL compared to the NICE guidelines.

However, some recent reports have raised concerns about the safety of EOSCAL. In a systematic review, Pettinger et al. (2019) (16) compared EOSCAL to NICE guidelines and observed that EOSCAL would have missed or delayed treatment in 14–22 cases out of 75 culture-proven EOS cases. Scott et al. (2021) (15) reviewed 31 consecutive cases of EOS among babies greater than 34 weeks' gestation who were admitted to the Royal Brisbane and Women's Hospital in Australia. They compared the efficacy of EOSCAL and the Queensland Health Guidelines in identifying EOS cases by 24 h of age. Using the neonatal calculator, 11 neonates (35%, 95% conﬁdence interval 19.2%–54.6%) would not have received antibiotics by 24 h of age. In comparison, only one neonate (3%, 95% conﬁdence interval 0.1%–16.7%) would not have received antibiotics by 24 h of age using the local guidelines. Similarly, Achten et al. (2021) (23) published a systematic review involving individual patient data meta-analysis of 234 cases of culture-proven EOS. The calculator recommended antibiotics for 40.6% (95% CI, 34.2%–47.2%) upon initial assignment and for 60.1% (95% CI, 54.5%–67.4%) by 12 h of age. However, this review included studies from different countries with varying baseline EOS risks and clinical practices, which introduces bias.

Our findings align with those of the above systematic reviews reported by Pettinger et al. (2019) (16) and Achten et al. (2021) (23). The finding that 40% of cases had no underlying risk factors and over a quarter of babies were asymptomatic at 12 h emphasizes the importance of continued postnatal observation.

Notably, the CDC recommendations performed better in the early identification and treatment of four of the eight babies who later expired. However, three of the expired babies were not identified by both methods, even at 12 h; despite that, these deaths occurred between 18 and 32 h of age.

While some studies suggest that babies exposed to chorioamnionitis have the highest risk of being missed by EOSCAL (16), our logistic regression found no significant predictors of missed cases, including chorioamnionitis. Although the proportion of chorioamnionitis was higher among the missed cases (46%) compared to the entire EOS cohort (28.5%), this difference did not reach statistical significance (*p* = 0.23), likely due to the limited sample size. Early use of intrapartum antipyretics is common in our settings. A previous study by our team observed that nearly half of the mothers with suspected chorioamnionitis had a peak intrapartum temperature below 38°C, stressing the possibility that antipyretics may affect the peak temperature (24). The developers of EOSCAL have also cautioned about the accuracy of peak intrapartum temperature in settings with routine antipyretic use in the delivery units.

In our team's prior quality improvement project using EOSCAL among asymptomatic term babies born to mothers with chorioamnionitis, none of the three EOS cases were missed (19). However, this protocol had additional safety nets, like excluding symptomatic and preterm babies and observing all babies for a minimum period of 48 h. Moreover, a higher baseline risk of 2 in 1,000 was used.

In summary, we observed that the direct application of EOSCAL to our setting would have been inferior to the existing practice. Antibiotic administration would have been delayed or missed in a significant proportion of EOS cases. Implementing the EOS Calculator should consider these proportions in local circumstances. Clinical vigilance remains essential for all newborns. Future studies should compare multiple strategies in local settings, involving careful monitoring and follow-up.

### Strengths and limitations

This study benefits from a large population base and consistent clinical management across the HMC, supported by high-quality electronic documentation that ensures the availability of all necessary data. Additionally, the sample size of 105 cases for external validation meets the recommended minimum of 100 cases suggested by Collins et al. (2016) (25), thereby providing a robust basis for validating the prognostic model.

The retrospective design of the study may introduce inherent biases. Furthermore, minor variations in baseline EOS risk over the seven-year study period could have influenced the predictive performance of the score.

The results are based on the 2017 version of EOSCAL. The updated version might perform differently. It should also be noted that this study does not provide a comprehensive evaluation of the calculator's overall performance; rather, the focus was specifically on its immediate ability to accurately identify neonates who subsequently developed positive blood cultures.

### Additional considerations related to the COVID-19 era

The study period (2015–2022) overlapped with the COVID-19 pandemic, which brought extensive challenges to perinatal care and may have indirectly influenced the risk of early-onset neonatal infections. Although maternal SARS-CoV-2 infection and vaccination were not recorded in our dataset, evolving viral lineages during this period exhibited marked geographic variation in transmissibility and immune evasion  (26). Recent advances in vaccine development, including novel circular RNA platforms, have demonstrated durable immune activation and strong translational potential for next-generation immunization strategies  (27).

Growing evidence also indicates that long-COVID can affect maternal health through sustained immune dysregulation, autoimmunity, and organ involvement, with potential consequences for pregnancy and neonatal outcomes  (28, 29). These persistent alterations in maternal physiology may influence placental function and neonatal immune priming, though further data are required to clarify causal relationships.

In parallel, the pandemic accentuated inherent diagnostic limitations in differentiating bacterial from viral or non-infectious inflammatory conditions. Studies examining the spectrum of fever of unknown origin have reaffirmed the complexity of pathogen determination and the evolving pattern of infectious etiologies across recent years  (30). These observations emphasize the necessity for robust, adaptive diagnostic approaches in neonatal infection surveillance, capable of maintaining high specificity even under global health system pressures.

Collectively, insights from the COVID-19 experience underscore the importance of strengthening infection-detection frameworks that remain resilient amid pandemics and adaptable to emerging pathogens.

## Conclusions

In this study, the incidence of early-onset sepsis was 0.88 per 1,000 live births, predominantly caused by Group B Streptococcus, consistent with trends reported in high-income countries. Notably, 40% of the affected neonates lacked identifiable prenatal risk factors, underscoring the critical need for vigilant postnatal clinical observation, as a substantial proportion of cases presented with delayed symptoms.

When comparing the EOSCAL to CDC guidelines, our findings indicate that EOSCAL would have delayed or missed antibiotic treatment in a significant number of cases, highlighting limitations of direct application in our setting. These results align with systematic reviews that question the safety of EOSCAL in certain populations. Therefore, local baseline risk, clinical practices, and rigorous monitoring protocols should inform the decision to implement EOSCAL alongside continued clinical vigilance for all newborns.

Future prospective studies comparing multiple risk stratification strategies within local contexts are needed to optimize EOS management while minimizing missed cases and unnecessary antibiotic use.

## Data Availability

The datasets presented in this study can be found in online repositories. The names of the repository/repositories and accession number(s) can be found below: Open Science Framework' via https://osf.io/m7drx/?view_only=90cd56351e5a46aab557919207a3bda1.

## References

[1] Weston EJ Pondo T Lewis MM Martell-Cleary P Morin C Jewell B The burden of invasive early-onset neonatal sepsis in the United States, 2005–2008. Pediatr Infect Dis J. (2011) 30(11):937–41. 10.1097/inf.0b013e318223bad2 21654548 PMC3193564

[2] Puopolo KM Benitz WE Zaoutis TE. Committee on fetus and newborn; committee on infectious diseases. Management of neonates born at ≥35 0/7 Weeks’ gestation with suspected or proven early-onset bacterial sepsis. Pediatrics. (2018) 142(6):e20182894. 10.1542/peds.2018-2894 30455342

[3] Schrag SJ Farley MM Petit S Reingold A Weston EJ Pondo T Epidemiology of invasive early-onset neonatal sepsis, 2005 to 2014. Pediatrics. (2016) 138(6):e20162013. 10.1542/peds.2016-2013 27940705

[4] Mackay CA Nathan EA Porter MC Shrestha D Kohan R Strunk T. Epidemiology and outcomes of neonatal sepsis: experience from a tertiary Australian NICU. Neonatology. (2024) 121(6):703–14. 10.1159/000539174 38889701 PMC11633889

[5] Sands K Spiller OB Thomson K Portal EA Iregbu KC Walsh TR. Early-onset neonatal sepsis in low- and middle-income countries: current challenges and future opportunities. Infect Drug Resist. (2022) 15:933–46. 10.2147/IDR.S294156 35299860 PMC8921667

[6] Dhudasia MB Mukhopadhyay S Puopolo KM. Implementation of the sepsis risk calculator at an academic birth hospital. Hosp Pediatr. (2018) 8(5):243–50. 10.1542/hpeds.2017-0180 29666161

[7] Mukhopadhyay S Dukhovny D Mao W Eichenwald EC Puopolo KM. 2010 perinatal GBS prevention guideline and resource utilization. Pediatrics. (2014) 133(2):196–203. 10.1542/peds.2013-1866 24446442 PMC3904275

[8] Uzan-Yulzari A Turta O Belogolovski A Ziv O Kunz C Perschbacher S Neonatal antibiotic exposure impairs child growth during the first six years of life by perturbing intestinal microbial colonization. Nat Commun. (2021) 12(1):443. 10.1038/s41467-020-20495-4 33500411 PMC7838415

[9] Reyman M van Houten MA Watson RL Chu MLJN Arp K de Waal WJ Effects of early-life antibiotics on the developing infant gut microbiome and resistome: a randomized trial. Nat Commun. (2022) 13(1):893. 10.1038/s41467-022-28525-z 35173154 PMC8850541

[10] Alm B Erdes L Möllborg P Pettersson R Norvenius SG Åberg N Neonatal antibiotic treatment is a risk factor for early wheezing. Pediatrics. (2008) 121(4):697–702. 10.1542/peds.2007-1232 18381533

[11] National Institute for Health and Care Excellence (NICE). Neonatal Infection: Antibiotics for Prevention and Treatment (NG195). London: NICE (2021). 34133110

[12] Canadian Paediatric Society. Management of suspected and proven bacterial sepsis of the newborn. Paediatr Child Health. (2017) 22(7):405–19.

[13] Kuzniewicz MW Puopolo KM Fischer A Walsh EM Li S Newman TB A quantitative, risk-based approach to the management of neonatal early-onset sepsis. JAMA Pediatr. (2017) 171(4):365. 10.1001/jamapediatrics.2016.4678 28241253

[14] Achten NB Klingenberg C Benitz WE Stocker M Schlapbach LJ Giannoni E Association of use of the neonatal early-onset sepsis calculator with reduction in antibiotic therapy and safety. JAMA Pediatr. (2019) 173(11):1032. 10.1001/jamapediatrics.2019.2825 31479103 PMC6724419

[15] Scott PA Lai M Inglis GDT Davies MW. Neonatal early-onset sepsis calculator safety in an Australian tertiary perinatal centre. J Paediatr Child Health. (2022) 58(5):863–7. 10.1111/jpc.15860 34990032

[16] Pettinger KJ Mayers K McKechnie L Phillips B. Sensitivity of the Kaiser Permanente early-onset sepsis calculator: a systematic review and meta-analysis. EClinicalMedicine. (2020) 19:100227. 10.1016/j.eclinm.2019.11.020 32140666 PMC7046522

[17] Thyvilayil SS Vellamgot AP Salameh K Kurunthattilthazhe SB Elikkottil A Dominguez LL Incidence and outcomes of neonatal group B streptococcal sepsis in Qatar—a multicentre study. BMC Pediatr. (2025) 25(1):41. 10.1186/s12887-025-05398-x 39825283 PMC11740512

[18] Verani JR McGee L Schrag SJ, Division of Bacterial Diseases, National Center for Immunization and Respiratory Diseases, Centers for Disease Control and Prevention (CDC). Prevention of perinatal group B streptococcal disease—revised guidelines from CDC, 2010. MMWR Recomm Rep. (2010) 59(RR-10):1–36. 21088663

[19] Vellamgot AP Salameh K Al-Bedaywi R Alhoyed SM Habboub L Abdellatif W Kaiser Permanente early-onset sepsis calculator as a safe tool for reducing antibiotic use among chorioamnionitis-exposed term neonates: Qatar experience. BMJ Open Qual. (2023) 12(4):e002459. 10.1136/bmjoq-2023-002459 37827729 PMC10582875

[20] Briggs-Steinberg C Roth P. Early-onset sepsis in newborns. Pediatr Rev. (2023) 44(1):14–22. 10.1542/pir.2020-001164 36587021

[21] Kuzniewicz MW Escobar GJ Forquer H Li S Shu D Kipnis P Update to the neonatal early-onset sepsis calculator utilizing a contemporary cohort. Pediatrics. (2024) 154(4):e2023065267. 10.1542/peds.2023-065267 39314183

[22] Yoshida R Piyasena C Galu S Thakkar D Battersby C. Regional observational study of missed cases of neonatal early-onset sepsis associated with the use of the SRC compared to NICE CG149. Arch Dis Child. (2022) 107:A176–7.

[23] Achten NB Plötz FB Klingenberg C Stocker M Bokelaar R Bijlsma M Stratification of culture-proven early-onset sepsis cases by the neonatal early-onset sepsis calculator: an individual patient data meta-analysis. J Pediatr. (2021) 234:77–84. 10.1016/j.jpeds.2021.01.065 33545190

[24] Vellamgot AP Salameh K AlBedaywi RR Habboub LH Daoud OA Atrash M Suspected clinical chorioamnionitis with peak intrapartum temperature <38°C: the prevalence of confirmed chorioamnionitis and short term neonatal outcome. BMC Pediatr. (2022) 22:381. 10.1186/s12887-022-03239-9 35410259 PMC8996607

[25] Collins GS Ogundimu EO Altman DG. Sample size considerations for the external validation of a multivariable prognostic model: a resampling study. Stat Med. (2016) 35(2):214–26. 10.1002/sim.6787 26553135 PMC4738418

[26] Choi JH Jun MS Jeon JY Kim HS Kim YK Jeon CH Global lineage evolution pattern of SARS-CoV-2 in Africa, America, Europe, and Asia: a comparative analysis of variant clusters and their relevance across continents. J Transl Infect Dis. (2023) 6(3):e0118. 10.2478/jtim-2023-0118 PMC10732492 38130632

[27] Xie J Ye F Deng X Tang Y Liang JY Huang X Circular RNA: a promising new star of vaccine. J Transl Infect Dis. (2023) 6(3):e0122. 10.2478/jtim-2023-0122 PMC10732498 38130633

[28] Guo M Shang S Li M Cai G Li P Chen X Understanding autoimmune response after SARS-CoV-2 infection and the pathogenesis/mechanisms of long COVID. Med Rev. (2024) 4(5):367–83. 10.1515/mr-2024-0013 PMC11495526 39444797

[29] Ewing AG Salamon S Pretorius E Joffe D Fox G Bilodeau S Review of organ damage from COVID and long COVID: a disease with a spectrum of pathology. Med Rev. (2024) 5(1):66–75. 10.1515/mr-2024-0030 PMC11834749 39974559

[30] Kang S Zheng R. Distribution of the causes of fever of unknown origin in China, 2013–2022. J Transl Infect Dis. (2024) 7(1):e0008. 10.2478/jtim-2024-0008 PMC11284625 39081273

